# Modiolus-Hugging Intracochlear Electrode Array with Shape Memory Alloy

**DOI:** 10.1155/2013/250915

**Published:** 2013-05-09

**Authors:** Kyou Sik Min, Sang Beom Jun, Yoon Seob Lim, Se-Ik Park, Sung June Kim

**Affiliations:** ^1^School of Electrical Engineering & Computer Science, Seoul National University, 1 Gwanak-ro, Gwanak-gu, Seoul 110-742, Republic of Korea; ^2^Department of Electronics Engineering, Ewha Womans University, 52 Ewhayeodae-gil, Seodaemun-gu, Seoul 120-750, Republic of Korea; ^3^Department of Brain & Cognitive Sciences, Ewha Womans University, 52 Ewhayeodae-gil, Seodaemun-gu, Seoul 120-750, Republic of Korea; ^4^Cognitive and Neural Systems, Boston University, 677 Beacon Street, Boston, MA 02215, USA

## Abstract

In the cochlear implant system, the distance between spiral ganglia and the electrodes within the volume of the scala tympani cavity significantly affects the efficiency of the electrical stimulation in terms of the threshold current level and spatial selectivity. Because the spiral ganglia are situated inside the modiolus, the central axis of the cochlea, it is desirable that the electrode array hugs the modiolus to minimize the distance between the electrodes and the ganglia. In the present study, we propose a shape-memory-alloy-(SMA-) embedded intracochlear electrode which gives a straight electrode a curved modiolus-hugging shape using the restoration force of the SMA as triggered by resistive heating after insertion into the cochlea. An eight-channel ball-type electrode array is fabricated with an embedded titanium-nickel SMA backbone wire. It is demonstrated that the electrode array changes its shape in a transparent plastic human cochlear model. To verify the safe insertion of the electrode array into the human cochlea, the contact pressures during insertion at the electrode tip and the contact pressures over the electrode length after insertion were calculated using a 3D finite element analysis. The results indicate that the SMA-embedded electrode is functionally and mechanically feasible for clinical applications.

## 1. Introduction

During the past several decades, cochlear implant systems have been established as a successful treatment for people suffering from severe hearing impairment due to hair cell loss. In general, a cochlear implant system consists of a multichannel intracochlear electrode, electronics in a hermetic package, and an external speech processor with a signal and power transmission coil. Briefly, the system converts sound signals into electrical signals and divides into multiple channels with different frequencies at the external speech processor. These signals then are radio-frequency- (RF-) modulated and transferred to the receiver/stimulator ASIC chip in a hermetic package through a transcutaneous coil link. After demodulation, the signals generate stimulating current pulses for each channel. Through the intracochlear electrode array, the current pulses stimulate ganglion cells in the inner ear, inducing auditory sensations in the recipients. The most important parts of the functionality of a cochlear implant are design of electrode array. Todays, insertion of a cochlear electrode array becomes far more important because it is required that newly developed electrode array can preserve residual hearing, be inserted deeper region of scala tympani, and stimulate targets more efficiently [1].

**Figure 1:**
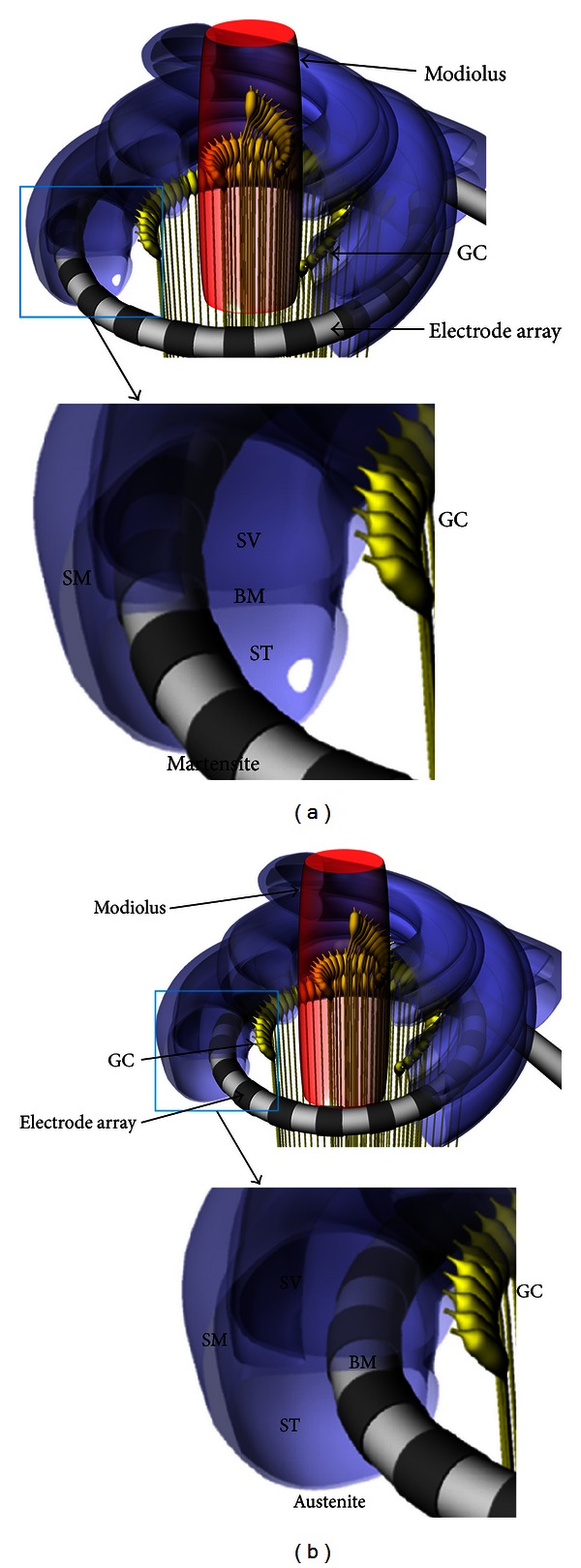
3D models of intracochlear electrode arrays inserted into the cochlea for stimulation on ganglion cells. (a) Straight intracochlear electrode without modiolus-hugging characteristics. (b) Modiolus-hugging intracochlear electrode (GC: ganglion cell, SM: scala media, SV: scala vestibuli, ST: scala tympani, and BM: basilar membrane).

One of the challenges to improve the current cochlear implant system is to realize spatially high-resolution electrical stimulation to restore sound perception close to normal hearing. To achieve this goal, it is essential to locate the intracochlear electrode array close to the spiral ganglia, the target of the electrical stimulation, because the current will diffuse and stimulate a large number of ganglia with low specificity if the electrode is located far from the target cells. In general, the electrode of a cochlear implant is inserted into the scala tympani because it has the largest cross-sectional area and an easy surgical operation site as shown in Figure 1. Stimulating currents flowing from the electrode site stimulate the ganglion cells located in the direction of the cochlear modiolus. Therefore, it is desired that the electrode is located close to the modiolus, as depicted in Figure 1(b), to reduce the distance between the electrode and the ganglia and increase the spatial specificity of the electrical stimulation [2]. When implanting the electrode array into the scala tympani, however, a straight shape is desirable to facilitate insertion by surgeons. If the electrode array is straight after molding with elastic silicon rubber, the electrode array is located along with the outer wall radius of the scala tympani due to the elastic restoration force of the electrode (Figure 1(a)).

Several strategies have been developed and employed for commercial cochlear implant systems to locate the electrode close to the modiolus. Advanced Bionics, Inc. and Cochlear Ltd. have developed modiolus-hugging electrodes using a preformed carrier which is held straight by an internal stylet before and during the insertion process [3]. After the partial insertion of the electrode and stylet, the electrode is pushed to its full insertion depth while holding the stylet at the same position. The electrode returns to its original spiral shape while being pushed toward the modiolus [4, 5]. Accordingly, the insertion depth of the stylet is critical in modiolus-hugging electrode array surgery. If the stylet is inserted far deeper into the cochleostomy, the electrode tip will come into contact with the cochlear outer wall, which may damage the spiral ligament or penetrate into the scala vestibuli. On the other hand, if the insertion depth of the stylet is too short, the apical curve of the electrode will curl before the first turn of the scala tympani [6]. Another method for modiolus-hugging was to insert an additional silastic structure called a positioner along the outside of the electrode after its insertion. The positioner pushes the electrode toward the modiolus, which locates the electrode sites in the vicinity of the ganglion cells [7]. However, when using this method, surgeons must undertake a precise insertion process twice. In addition, it was reported that the enlarged hole for the additional insertion of the positioner can cause meningitis [8].

In order to overcome these disadvantages, it was proposed to make the stylet with shape memory alloy (SMA), which converts its initial straight shape into a curved shape at the body temperature [9]. With this method, the electrode hugs the modiolus once it is inserted into the scala tympani by the restoration force of SMA stylets, during which the modiolus-hugging shape is memorized. However, it is not easy to pull out the electrode or to reinsert it once it is inserted because the SMA force always exists at the temperature of the human body. The heat from the surgeon's hands may also trigger SMA transformation before the insertion of the electrode.

In the present study, we propose an intracochlear electrode embedded with SMA without the drawbacks related to the transition temperature of the human body. Similar to the previous method, the SMA is pretreated to have a modiolus-hugging shape above its transition temperature. However, because the SMA is transformed into its memorized shape slightly above the body temperature, the electrode can be handled by a surgeon without SMA transformation during the insertion process. The transformation into the modiolus-hugging shape can be controlled by heating via an electrical current to the SMA after the insertion. We developed an eight-channel intracochlear electrode in which SMA wire is embedded. A finite element analysis was performed to verify the mechanical safety of the electrode array compared with conventional electrode arrays.

## 2. Materials and Methods

### 2.1. Shape Memory Alloy

**Figure 2:**
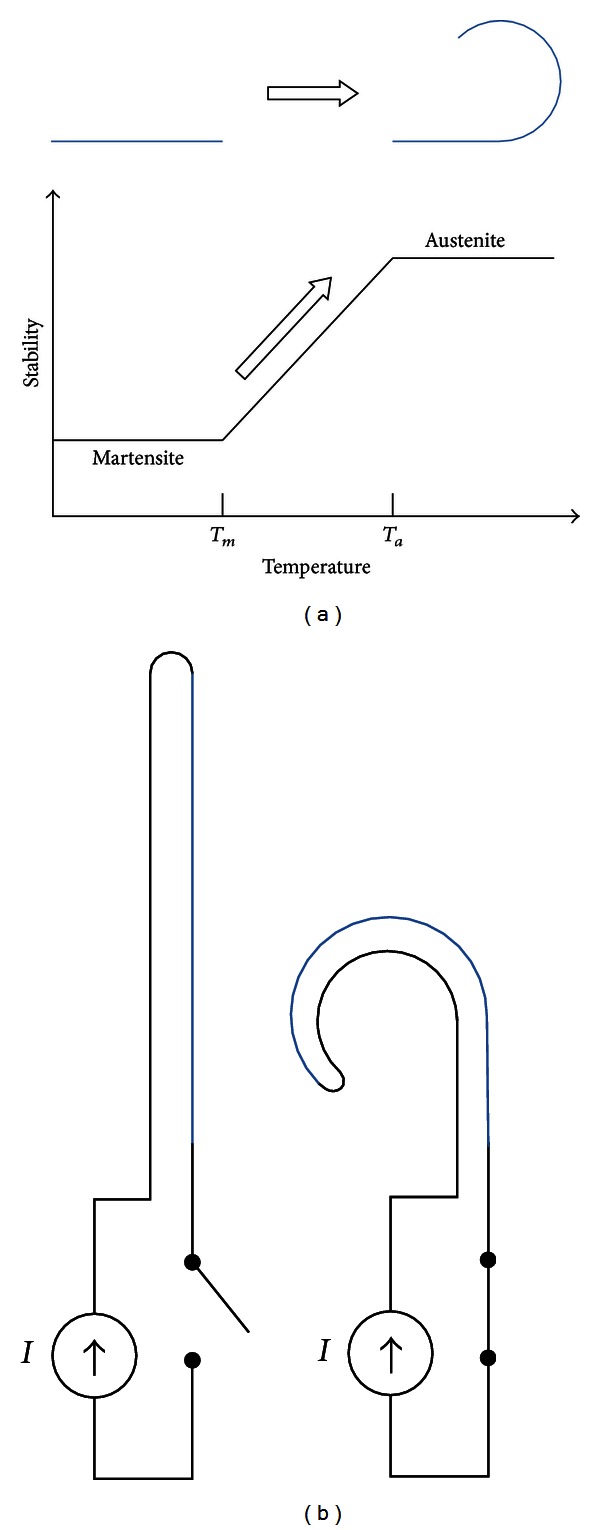
Transformation of shape memory alloy (SMA): (a) SMA changes its straight shape (martensite) into the modiolus-hugging shape (austenite), (b) the SMA transformation is triggered by resistive heating of electrical current.

The shape memory alloy (SMA) has two different phases: martensite and austenite. Martensite is the relatively soft and easily deformable phase of shape memory alloys, and it exists at a lower temperature. Austenite, the stronger phase of shape memory alloys, occurs at higher temperatures. Above a specific temperature, the deformed SMA in martensite is transformed into the austenite phase, which is configured as the original shape of the wire. The temperatures at which the transformation begins and ends are defined as *T*_*m*_ and *T*_*a*_ (Figure 2), respectively. In this study, *T*_*m*_ is defined as higher than the temperature of the human body in order to prevent the SMA from being transformed by heat from the surgeon's hands or by the patient's body while inserting the electrode into the cochlear scala tympani. *T*_*a*_ is defined as low as possible so as not to cause tissue damage by the heat generated from the SMA. Using titanium-nickel alloy, the SMA is prepared such that *T*_*m*_ is 40°C and *T*_*a*_ is 45°C (Jin-Sung Ltd., Eui Wang, Korea).

**Figure 3:**
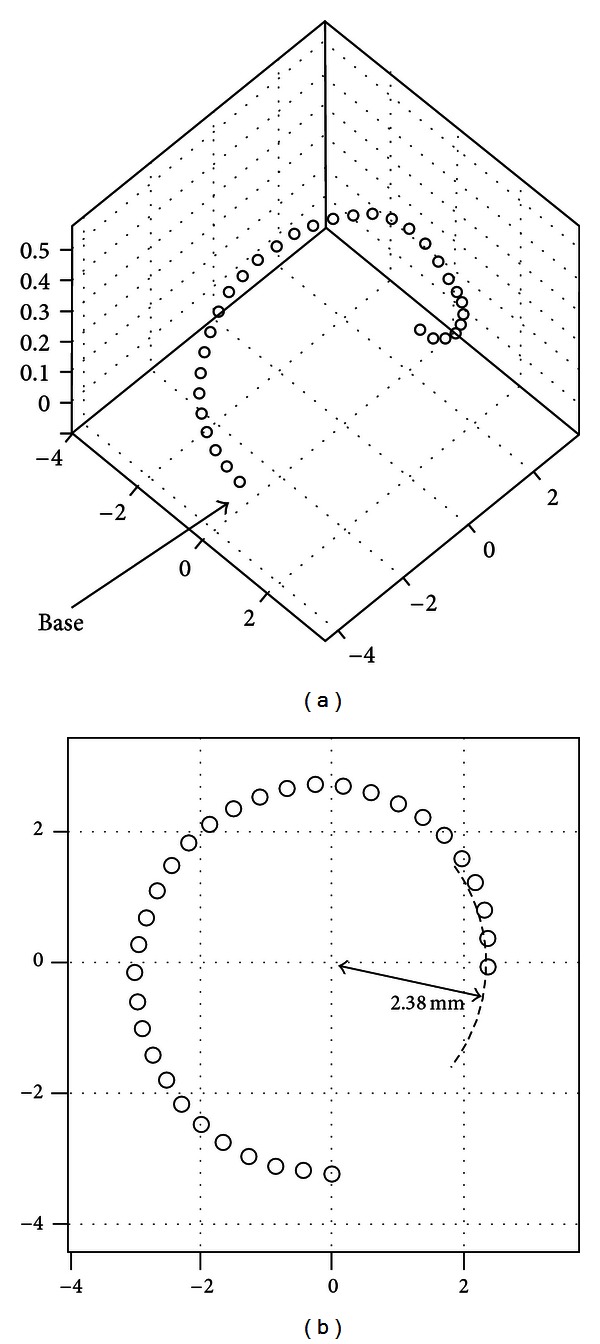
Trace of electrode tip during the insertion into scala tympani of human cochlea (all units are mm in this picture): (a) 3D trajectory of the electrode tip and (b) the trajectory of the electrode tip projected on *x*-*y* plane.

The human cochlear canal is a spiral structure with 2.5 turns. If straightened, its length is approximately 32 mm from the base to the apex of the cochlea. However, the maximum insertion angle of the intracochlear electrodes is usually 360° and less than 25 mm in length, as shown in the modeling result of the trace of the electrode tip in Figure 3. In Figure 3(b), the smallest radius of curvature of the trace is 2.38 mm. To simplify the fabrication of SMA wires to be embedded in electrodes, the displacement on the *z*-axis (0.336 mm) was ignored because it is much smaller than those of the *x*- and *y*-axes, as depicted in Figure 3. The SMA wires (0.1 mm diameter) were fabricated in the shape of a spring whose curvature is 4.7 mm in diameter so that it can ensure that the curvature hugs the modiolus. The SMA spring was cut to have a length of 15 mm and an angle of 360°.

### 2.2. Electrode Fabrication and Transformation Test

**Figure 4:**
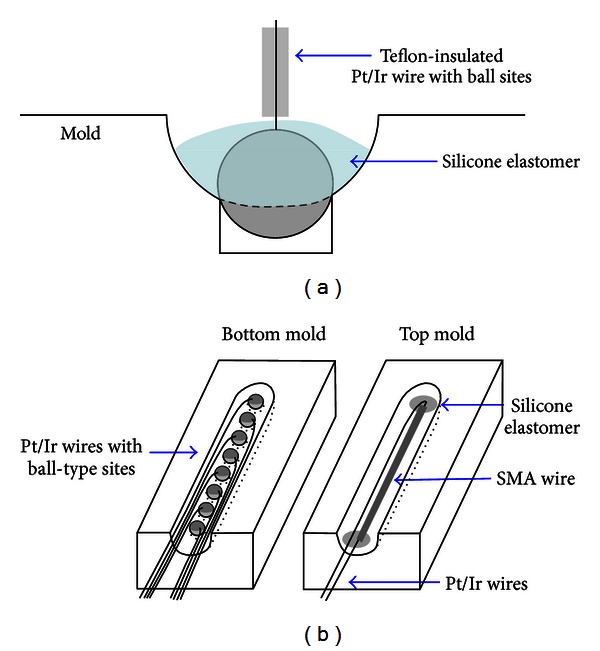
Fabrication of the SMA-embedded ball-type intracochlear electrode: (a) ball site fixation at a hole in the bottom mold and (b) fixation of eight-channel ball electrodes and the SMA wire at bottom and top molds, respectively.

An eight-channel intracochlear electrode array was fabricated. In this process, the ball-shaped sites are made from Teflon-coated 25 *μ*m (dia.) 90% Pt/10% Ir-alloy wire (A-M systems, Inc., USA) by melting the wire with an oxygen/acetylene minitorch. The diameter of the ball sites is 420 *μ*m. The balls are fixed at a hole in the bottom mold, as depicted in Figure 4, using silicone elastomer MED 4211 (Nusil, Ltd., USA). Both ends of the SMA wire are soldered with the same wires as the electrodes. The SMA wire is straightened and is then fixed at the top mold with the elastomer. After joining two molds, degassed silicone elastomer is injected into the inlet. To prevent the SMA from being transformed, the elastomer is cured at room temperature for 2 days and then at 150°C for 30 minutes to complete the curing. The distance between adjacent sites is 1.8 mm. The total length of the electrode to be inserted into the cochlea is 21.5 mm. The electrode diameters were 0.6 mm at the apex and 0.8 mm at the base.

To determine if the fabricated SMA-embedded electrode array is transformed into the modiolus-hugging shape, various amplitudes of electrical current are applied to wires connected to both ends of the SMA. The electrode is also inserted into a clear human cochlear model, and the restoration force of the SMA triggered by resistive heating is applied to verify that the electrode can change its shape into a modiolus-hugging shape.

### 2.3. Electrode Insertion Simulation

It was reported that the insertion of a cochlear electrode may cause trauma at the basilar membrane, which is very thin and soft [10–12]. Researchers inserted electrodes into the cochleae of human cadavers or the cochleae of animals in an effort to prove the mechanical safety of the electrodes [13–15]. It is also known that the trauma can be predicted by the stiffness of the electrodes [6]. We performed a finite element analysis on the pressure generated by electrode insertion using a three-dimensional simulation (ANSYS 14 Workbench, ANSYS, Inc., USA). The three-dimensional human cochlear model is reconstructed using Rhinoceros (Robert McNeel & Associates, USA). First, a human scala tympani image is used for the base model of the cross-section of the entire scala tympani. The outline of the cross-section of the scala tympani is arranged along a curve defined in cylindrical coordinates as follows:

(1) $$\begin{array}{c} R=1.14987e^{0.075458\varphi },\\ h=3.23203e^{-0.126636\varphi }, \end{array}$$

where *R* is radius from the central axis of cochlear spiral, *h* is height along the spiral axis and *φ* is cochlear angle (0~5*π*).

The patterns are then rescaled so that the areas fit cross-sectional areas along the depths [16]. The centers of the mass of the rescaled patterns are positioned along the aforementioned cochlear curve. The surface of the scala tympani is generated by connecting the closed curves. The electrode is modeled with diameters of 0.6 mm at the apex and 0.8 mm at the base. Eight platinum-iridium wire models are linearly arranged along the *z*-axis, which is parallel to the modiolus. The nitinol (Ti-Ni alloy) wire is modeled at the bottom of the platinum-iridium wire arrays.  Table 1 depicts the material properties used in the finite element analysis. Because the SMA-embedded electrode array is designed to be inserted under the martensite temperature (*T*_*m*_), the mechanical parameters of martensite are used for the SMA. The stress-strain value of the SMA-embedded electrode can be expressed by the following equation using the nonlinear uniaxial stress-total strain relationship [17]:

(2) σ(ε)=(Ec1+(Ecε/2fc)2   +Es1+(Esε/2fs)2+∑Ew1+(Ewε/2fw)2)ε,

where *σ* = stress at strain *ε*, *E*_*c*_, *E*_*s*_, *E*_*w*_ = Young's modulus of silicone elastomer, SMA, and Pt/Ir wire, respectively, and *f*_*c*_, *f*_*s*_, *f*_*w*_ = the ultimate compressive strengths of silicone elastomer, SMA, and Pt/Ir wire, respectively.

Based on the stress-strain formula, the pressure at the electrode tip during the insertion process is calculated because insertion trauma tends to occur at the interface of the electrode tip and the cochlear tissue. The pressure over the electrode is also simulated by a time-lapse method. After completing the insertion process, the contact pressures are obtained along the electrode length. The displacement and fixed constraints are defined at the base plane of the electrode array and the scala tympani, respectively. The electrode array is inserted from the center of the cross-section of the scala tympani at 80°. The electrode array model is rotated by 10° on the *z*-axis. The electrode model is inserted to 18 mm, which is 360° in terms of the cochlear angle. In the analysis, the stress intensity at the interface of the electrode is calculated. 

## 3. Result

**Figure 5:**
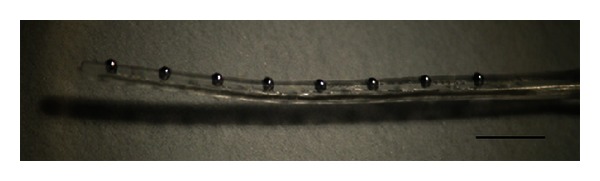
Eight-channel SMA-embedded intracochlear electrode array (scale bar: 2 mm).

**Figure 6:**
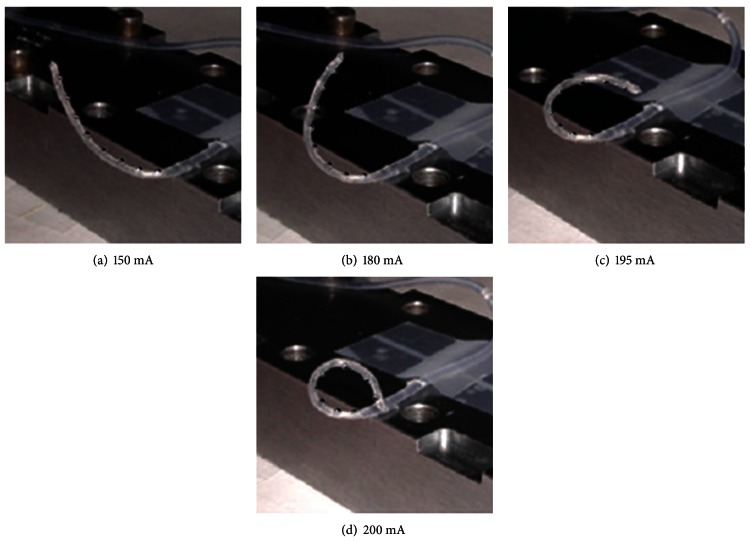
Electrode transformation induced by resistive heating of electrical current through the embedded SMA wire.

The SMA-embedded eight-channel electrode array is fabricated as shown in Figure 5. The SMA wire is seen through the transparent silicone elastomer envelope. The electrical current through the SMA wire is increased while observing the electrode's shape. The electrical current-generated resistive heat at the SMA begins to induce the transformation of the electrode shape from a current level of approximately 150 mA, as shown in Figure 6(a). When the current level was increased to 208 mA, the transformation was completed by changing the electrode into the modiolus-hugging shape as memorized in the SMA wire (Figure 6(d)). This result indicates that the SMA phase is shifted from martensite to austenite by the resistive heat generated by the electrical current. During this heating process, the maximum power dissipation was 75 mW.

**Figure 7:**
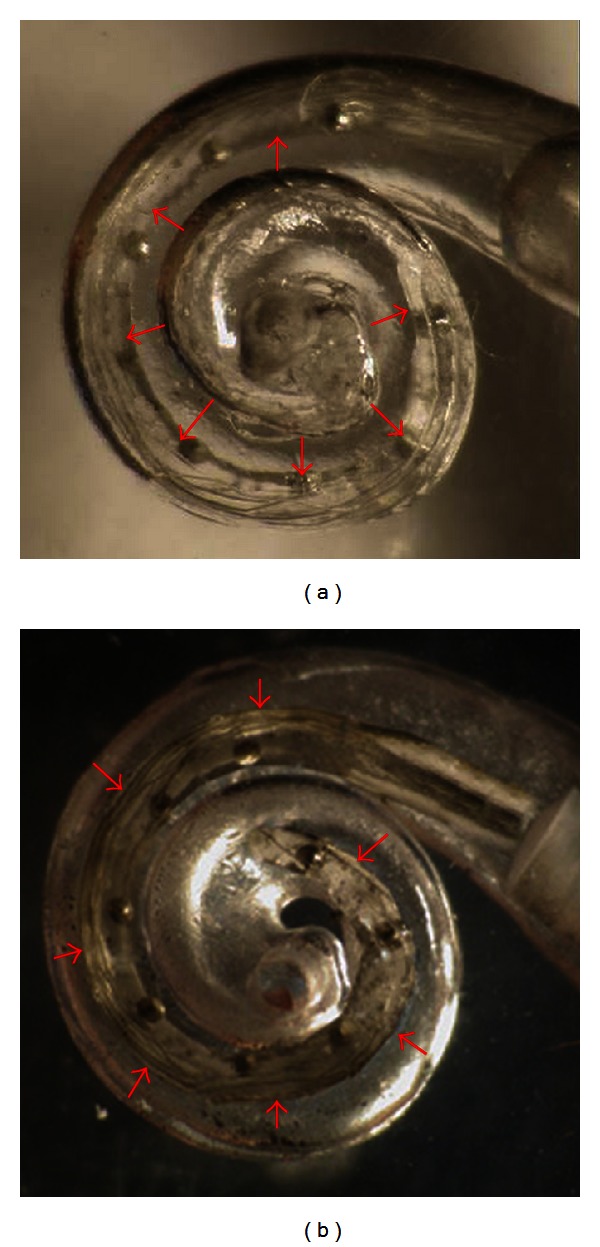
Insertion test into a transparent plastic cochlear model: (a) electrode without SMA: (b) electrode with a modiolus-hugging SMA.

The electrode without SMA is located along the outer wall of the transparent scala tympani model (Figure 7(a)). For an electrode with an SMA stylet, the electrode hugs the central *z*-axis of the model, as shown in Figure 7(b), after applying electrical current through the SMA. In order to mimic an actual surgical situation, the cochlear model was filled with lubricant prior to the insertion of the electrode [2].

**Figure 8:**
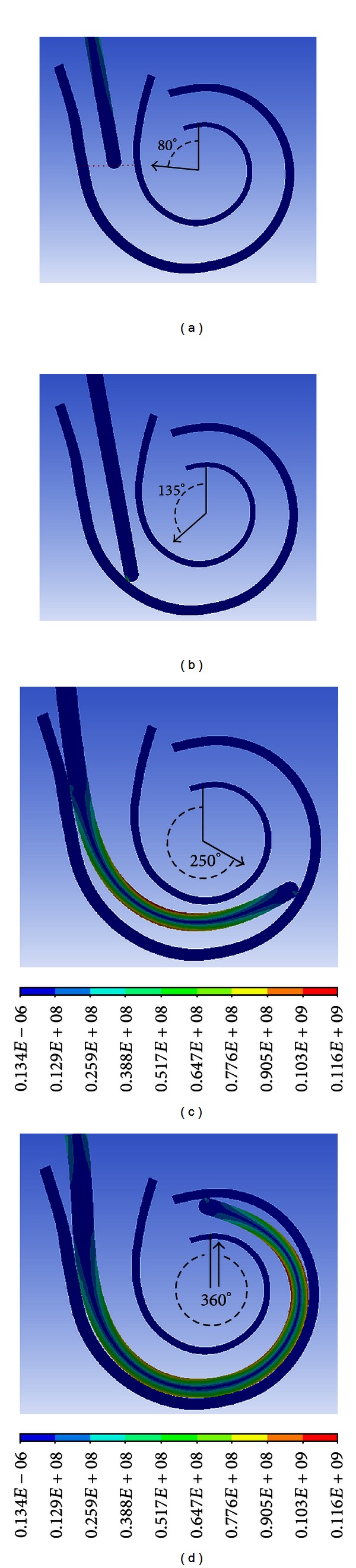
Cross-sectional views of 3D simulation of pressure distribution on the electrode during electrode insertion into cochlea.

The trajectories of the electrode array with the SMA are visualized in a 2D cross-section of the scala tympani (Figure 8). The insertion process begins from the location with a cochlear angle of 80° (Figure 8(a)) and is performed up to 18 mm in depth representing a cochlear angle of 360°. The electrode initially came into contact with the wall of the cochlea at a cochlear angle of 135° when it was inserted to a depth of 6 mm. As it was inserted further into the cochlea, the electrode continued to bend and conform to the curvature of the cochlear outer wall. The insertion was finished when the tip reached the position with a cochlear angle of 360° (Figure 8(d)) and a total insertion depth of 18 mm. The stress applied to the electrode is shown in different colors in Figure 8. 

**Figure 9:**
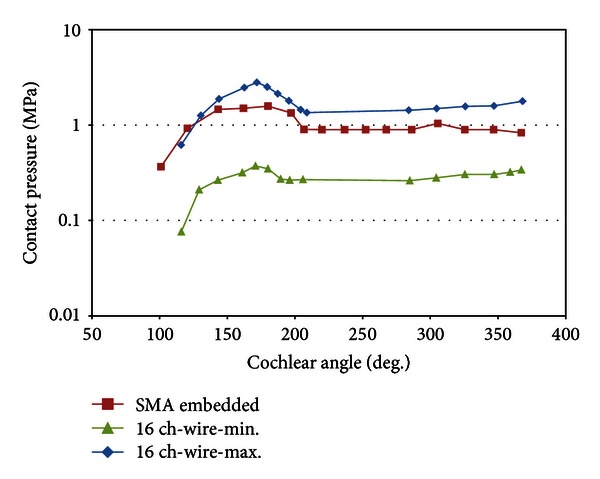
Contact pressures at the electrode tip during insertion.

**Figure 10:**
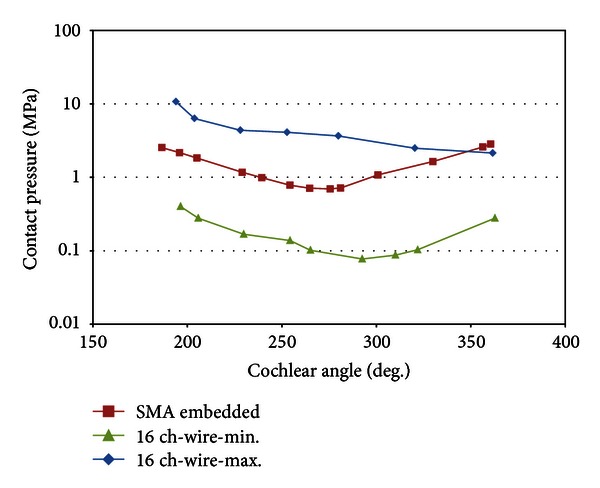
Contact pressure distributions along the length of the electrode after insertion.

In order to verify the feasibility of SMA-embedded electrodes, two mechanical parameters are evaluated using a finite element analysis. First, the contact pressures at the electrode tip during insertion are shown in Figure 9. Second, after the insertion is completed, the contact pressure distribution is calculated along the length of the electrode array and shown in Figure 10. To compare conventional electrode arrays, for both tests, 16-channel electrode arrays are employed with different arrangements of wires. Previously, it was reported that the maximum and the minimum contact pressure levels occur when the wires in the electrode are assembled along the vertical and horizontal planes, respectively [16]. The contact pressures at the electrode tip are evaluated because it was previously reported that most traumatic events arose between the electrode tip and basilar membrane.

As shown in Figure 9, the contact pressure increased steeply after the electrode tip initially came into contact with the inner wall at a cochlear angle of approximately 100°. The maximum value of the contact pressure is 1.58 MPa during insertion at 180°. It decreased and converged to 0.9 MPa during the rest of insertion process. In terms of the electrode position and deformation, this result indicates that the maximum contact pressure at the electrode tip is attributed to the initial bending of the straight electrode. Moreover, the pressure becomes constant once the electrode bends to fit to the curvature of the cochlear outer wall from its original straight shape. After the completion of the electrode insertion process, as depicted in Figure 10, the maximum pressure occurs approximately at 200°, where the deformation begins. The pressure decreased from 200° to 275° and increased again to the apical region of the electrode.

## 4. Discussion

 In this study, we developed an eight-channel, SMA-embedded, intracochlear electrode. The intracochlear electrode could be located in the vicinity of spiral ganglion cells via the formation of a modiolus-hugging shape of the SMA. When using this method, the electrode maintains a straight shape during the insertion process and then transforms into a curved shape which can surround the cochlear modiolus. This proposed method reduces the distance between stimulation electrode sites and the targeted spiral ganglion cells, which enables an effective stimulation with lower power while increasing the operation time using a battery. Moreover, the discrimination between channels is also improved due to the reduced amount of current spreading. The proposed intracochlear electrode has advantages in that it does not produce a vacant lumen due to the stylet removal process, creating a possible infection route, and does not require the insertion of an additional insert structure to fill the cavity of the scala tympani, which has been used commercially for modiolus-hugging.

On the other hand, there are other factors to consider. There is a possibility that the heat generated by the electrical current can damage the tissue surrounding the electrodes. However, the transition temperature was adjusted from 40°C to 45°C, which is close to the body temperature, and the SMA wire is insulated by thick silicone rubber. Though not proved in this paper, redundant heat accumulation can be avoided by applying current pulses instead of a constant current flow. Electrical safety should be also considered. In this study, the transformation of the SMA required about 200 mA of DC electrical current. This amount was much higher than the stimulation current, with its maximum amplitude of only several mA. In order to avoid stimulating adjacent cells with this large amount of current, it should be ensured that the entire current path is perfectly insulated properly at least during the implantation process. Using the proposed technique, advanced intracochlear electrodes with segmented SMAs can also be developed. If each SMA segment is converted to a curved shape of the martensite phase in an orderly process from the apex to the base, electrode insertion into the scala tympani can be done with little insertion force. In addition, even after complete insertion, the position of the electrode can be adjusted by applying electrical current through specific SMA segments if necessary [22].

In this study, three-dimensional modeling of the scala tympani is done using a cross-sectional outline of an actual cochlear image to calculate the contact pressure during and after electrode insertion. This is a simplified model of the human cochlea because the cochlear duct does not consist of only the scala tympani. Although the scala tympani model is sufficient to draw a conclusion about general insertion safety, a full cochlear model is required to analyze complicated failure modes. To generate a realistic cochlear model, the cross-sections should be reconfigured including the basilar membrane and other canals such as the scala media and scala vestibuli. 

The simulation results indicate that the eight-channel SMA-embedded electrode has a comparable stiffness as 16 channel cochlear electrode which was reported previously [16]. The stiffness of the intracochlear electrodes is one of the most important parameters determining the safety of electrode insertion, as a stiffer electrode incurs greater risk of penetrating through the basilar membrane during the insertion process. Using the typical method of cochlear electrode fabrication of molding metal wires with a silicone rubber carrier, the stiffness of the electrode is determined by the number and the diameter of the metal wires. In this study, to show the feasibility of the modiolus-hugging SMA-embedded electrode, an electrode with eight channels is fabricated instead of more than 10 channels. In order to achieve a SMA-embedded electrode with more channels, the diameter of the SMA wire needs to be increased to ensure sufficient restoration force, or the diameters of the individual metal wires should be decreased. In addition, the overall stiffness of the electrode needs to be within a range such that safe insertion is ensured without damage to the cochlea. 

As an alternative method to realize a high-density cochlear electrode array with SMA, a micropatterned electrode array based on thin film can be employed [23]. Using this method, a SMA-embedded electrode can be fabricated without a reduction of the number of channels because the thin-film-based electrode can have a much larger number of channels without increasing the stiffness of the electrode, unlike a wire-based electrode array. However, the mechanical insertion behavior of an electrode based on a thin-film array should be evaluated, including the stiffness, insertion depth, and rotation of the electrode tip in an *in vitro* study or via a simulation.

 To employ this method in clinical cochlear implant procedures, the biocompatibility of the SMA material should be proven during long-term implantation assessments. Due to the superelasticity and the large deformation capability of TiNi SMA, it has been used for several medical applications, such as orthodontic arch wires; orthopedic implants; and stents for coronaries, esophagi, or large intestines. Despite these applications, the toxicity of TiNi alloys has remained controversial for long-term applications due to the high nickel content. However, recent studies have reported that TiNi can be implanted into the human body for long-term use because an oxidized layer of TiO_2_ on the TiNi surface prevents the possible loosening of nickel. This type of passivated TiO_2_ layer is known to be very stable chemically and mechanically [24]. Therefore, TiNi SMA is expected to be used in regular medical applications such as cochlear implants in the near future [25, 26]. 

## Figures and Tables

**Table 1:** Mechanical parameters of human cochlea and materials of intracochlear electrodes.

Material	Elastic modulus	Poisson's ratio
Bone (cochlear inner wall) [18]	20 GPa	0.3
Pt/Ir/PTFE [19]	8.27 GPa	0.3
Silicone elastomer [20]	0.45 MPa	0.5
TiNi (martensite) [21]	28 GPa	0.33

## References

[1] Rebscher SJ, Hetherington A, Bonham B, Wardrop P, Whinney D, Leake PA (2008). Considerations for design of future cochlear implant electrode arrays: electrode array stiffness, size, and depth of insertion. *Journal of Rehabilitation Research and Development*.

[2] Rebscher SJ, Heilmann M, Bruszewski W, Talbot NH, Snyder RL, Merzenich MM (1999). Strategies to improve electrode positioning and safety in cochlear implants. *IEEE Transactions on Biomedical Engineering*.

[3] Eshraghi AA, Yang NW, Balkany TJ (2003). Comparative study of cochlear damage with three perimodiolar electrode designs. *Laryngoscope*.

[4] Pasanisi E, Vincenti V, Bacciu A, Guida M, Bacciu S (2002). The nucleus contour electrode array: an electrophysiological study. *Laryngoscope*.

[5] Treaba C-GW, Dadd F, Darley DI, Parker JL (2002). Cochlear implant electrode array.

[6] Rebscher SJ, Hetherington A, Bonham B, Wardrop P, Whinney D, Leake PA (2008). Considerations for design of future cochlear implant electrode arrays: electrode array stiffness, size, and depth of insertion. *Journal of Rehabilitation Research and Development*.

[7] Yang NW, Hodges AV, Balkany TJ (2000). Novel intracochlear electrode positioner: effects on electrode position. *The Annals of Otology,Rhinology & Laryngology*.

[8] Arnold W, Bredberg G, Gstöttner W (2002). Meningitis following cochlear implantation: pathomechanisms, clinical symptoms, conservative and surgical treatments. *Journal for Oto-Rhino-Laryngology and its Related Specialties*.

[9] Dadd F, Darley DI, Gibson P, Parker JL, Treaba C (2007). Double stylet insertion tool for a cochlear implant electrode array.

[10] Kennedy DW (1987). Multichannel intracochlear electrodes: mechanism of insertion trauma. *Laryngoscope*.

[11] Welling DB, Hinojosa R, Gantz BJ, Lee JT (1993). Insertional trauma of multichannel cochlear implants. *Laryngoscope*.

[12] Roland PS, Wright CG (2006). Surgical aspects of cochlear implantation: mechanisms of insertional trauma. *Advances in Oto-Rhino-Laryngology*.

[13] Eshraghi AA, Yang NW, Balkany TJ (2003). Comparative study of cochlear damage with three perimodiolar electrode designs. *Laryngoscope*.

[14] Wardrop P, Whinney D, Rebscher SJ, Roland JT, Luxford W, Leake PA (2005). A temporal bone study of insertion trauma and intracochlear position of cochlear implant electrodes. I: comparison of nucleus banded and nucleus Contour*™* electrodes. *Hearing Research*.

[15] Wardrop P, Whinney D, Rebscher SJ, Luxford W, Leake P (2005). A temporal bone study of insertion trauma and intracochlear position of cochlear implant electrodes. II: comparison of spiral clarion*™* and hiFocus II*™* electrodes. *Hearing Research*.

[16] Lim YS, Park SI, Kim YH, Oh SH, Kim SJ (2005). Three-dimensional analysis of electrode behavior in a human cochlear model. *Medical Engineering and Physics*.

[17] Kachlakev D, Miller T, Yim S, Chansawat K, Potisuk T (2001). Finite element modeling of reinforced concrete structures strengthened with FRP laminates. *Final Report SPR*.

[18] Baker GJ, Steele CR, Tolomeo JA, Zetes-Tolometo DE (2000). Cochlear mechanics. *The Biomedical Engineering Hanbook*.

[19] McNaughton TG, Horch KW Mechanical testing of metallic and polymeric intrafascicular electrodes.

[20] van Krevelen DW, te Nijenhuis K (2009). *Properties of Polymers: Their Correlation with Chemical Structure; Their Numerical Estimation and Prediction from Additive Group Contributions*.

[21] contributors W (2013). Nickel titanium. http://en.wikipedia.org/w/index.php?title=Nickel_titanium&oldid=535331827.

[22] Jun SB, Kim SJ (2002). Fabrication method for intracochlear electrodes.

[23] Kim JH, Min KS, Lee HS, Park MH, Oh SH, Kim SJ A polymer based intracochlear electrode for low cost, but highly effective cochlear implantation.

[24] Filip P, Lausmaa J, Musialek J, Mazanec K (2001). Structure and surface of TiNi human implants. *Biomaterials*.

[25] Lipscomb IP, Nokes LDM (1996). *The Application of Shape Memory Alloys in Medicine*.

[26] Wu MH, Hodgson DE, Biermann RJ (1990). Shape memory alloys. *Metals Handbook*.

